# Acute popliteal thrombus following total knee arthroplasty: A case report

**DOI:** 10.1097/MD.0000000000022500

**Published:** 2020-10-16

**Authors:** Nicholas Runge, Lauren Hollifield, Margaret Arnold, Julius Oni

**Affiliations:** a Alabama College of Osteopathic Medicine, Dothan, AL; b University of Nevada Las Vegas School of Medicine, Las Vegas, NV; c Johns Hopkins University School of Medicine, Baltimore, MD.

**Keywords:** popliteal thrombus, total knee arthroplasty

## Abstract

**Introduction:**

We report the youngest documented patient (38 years old) to develop an acute popliteal artery thrombus following primary total knee arthroplasty (TKA).

**Patient Concerns:**

The patient presented for an elective TKA secondary to posttraumatic arthritis. Past medical history included a tibial plateau fracture, two knee arthroscopies and an elevated body mass index (37.53). A right TKA was performed with no intraoperative complications. Two hours postoperatively, the right foot was poikilothermic and lacking dorsalis pedal pulse.

**Diagnosis:**

Popliteal artery thrombus confirmed by angiogram and venous duplex. Interventions: Immediate vascular surgery consult and subsequent embolectomy.

**Outcomes:**

At 1 year postoperatively, the patient is doing well with no further complications.

**Conclusion:**

Due to the lack of significant past medical history putting this patient at risk, future research should focus on prior trauma, age, and BMI as risk factors, specifically in patients undergoing TKA.

## 1 Introduction

Total knee arthroplasty (TKA) is a common treatment for severe end-stage knee osteoarthritis. Some of the most common complications after TKA include infection, dislocation/fracture, and deep venous thrombosis (DVT).^[1]^ Albeit rare (0.057%), a thrombus associated with TKA may occur in the popliteal artery.^[2,3]^ Prompt recognition of popliteal artery occlusion or transection is required to prevent loss of limb or death.^[4]^ Maximal care should be taken to avoid the popliteal artery during knee surgery, especially TKA. Thrombotic events are associated with trauma or fractures, major orthopedic surgery, hypercoagulability, increased age, and metabolic syndrome.^[5]^ In the following case, we report the youngest documented patient to develop an acute popliteal artery thrombus following primary TKA.

## 2 Patient information

A 38-year-old Caucasian male presented for a right total knee arthroplasty (TKA) at Johns Hopkins Bayview Medical Center. He elected to undergo primary TKA, secondary to end-stage posttraumatic osteoarthritis that was refractory to non-operative treatment. The patient was a non-smoker with no known significant past medical history, other than a body mass index (BMI) of 37.53. His past surgical history was significant for open reduction internal fixation (ORIF) of a right tibial plateau fracture in 2009, and subsequent right knee arthroscopies in May 2010 and June 2011. Due to persistent pain and symptoms after exhausting conservative treatment, he wished to proceed with surgical intervention. A staged hardware removal followed by TKA was recommended. The hardware removal was performed without complications, and the TKA was planned for 3 months later.

## 3 Findings

At the preoperative TKA appointment, the incision from the hardware removal was well healed. Physical examination demonstrated range of motion from 0° to 90°of flexion. Neutral alignment and good stability were noted. Neurovascular status was intact, distally.

## 4 Diagnostic assessment

Preoperative testing for inherited and acquired thrombophilia disorders were not indicated due to his benign past medical history and no indication of a family history of protein C or S deficiency, Factor V Leiden or other acquired deficiencies that promote coagulation. Preoperative x-rays (A and B), prior to hardware removal from previous ORIF, demonstrate retained hardware with advanced posttraumatic arthritis. Radiographs taken at the preoperative appointment prior to the TKA revealed severe medial joint space narrowing with associated osteophytes and subchondral sclerosis (A and B). He elected to proceed with primary TKA.

**Figure 1:**
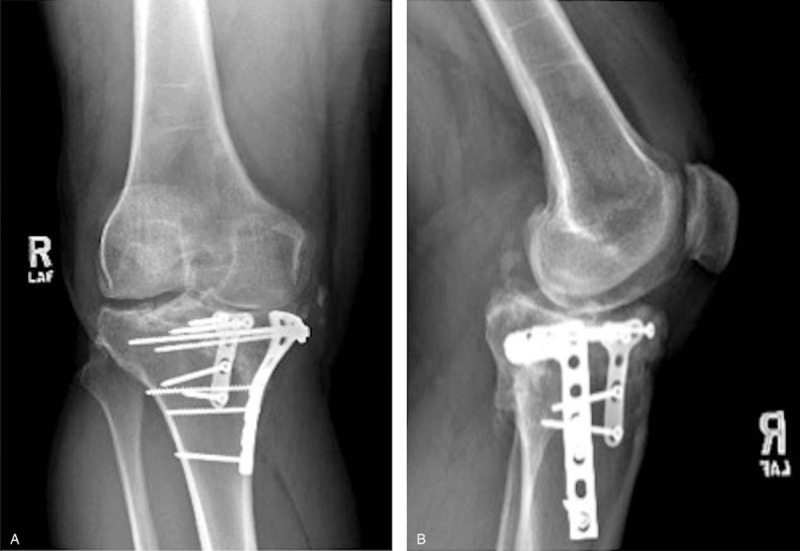
1A: Preoperative AP x-ray demonstrating retained hardware from previous tibial plateau ORIF performed in 2009. 1B: Lateral x-ray of Figure 1A.

**Figure 2:**
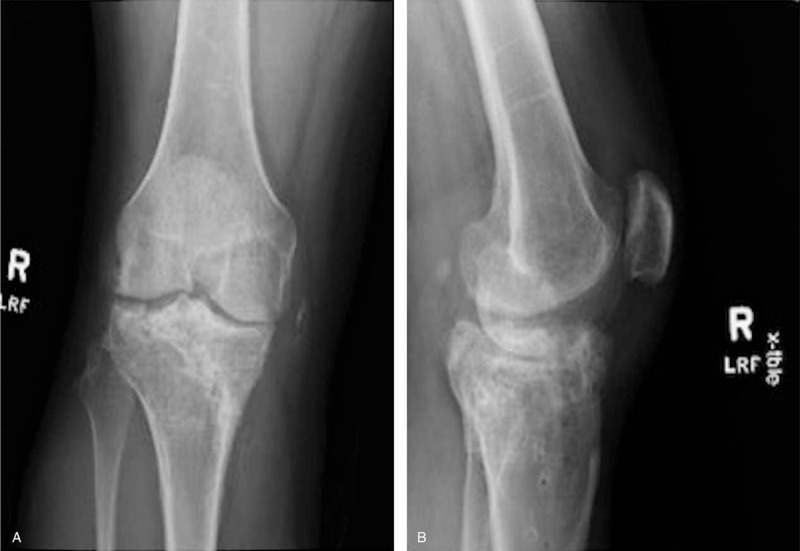
2A: AP preoperative x-ray demonstrating hardware removal, and end-stage posttraumatic arthritis. 2B: Lateral x-ray of Figure 2A.

## 5 Therapeutic intervention

A board certified, fellowship trained, joint replacement specialist performed a cruciate retaining R-TKA under spinal anesthesia. Two grams of Ancef were given 30 minutes prior to the operation. Zimmer-Biomet Nex Gen CR Flex primary knee (Warsaw, IN) implants were used. Blood loss was minimal (100 mL). Tourniquet time was less than 60 minutes. There were no apparent intraoperative complications but the patient had significant preoperative stiffness, which made soft tissue mobilization slightly more difficult throughout the case. Postoperative radiographs demonstrated excellent alignment and fixation of the femoral and tibial components (A and B). Two hours postoperatively, the patient developed increased right anterior shin pain and lower leg swelling. The patient's right lower extremity gross sensation was intact but overall diminished, compared to the left. Left lower limb pulses were 2+; however, the right posterior tibial and dorsalis pedis pulses were non-palpable and undetectable via Doppler ultrasound. CT Angiography with contrast and arterial duplex ultrasound revealed a thrombus in the right distal popliteal artery, inferior to the joint line (Figs. 4(A and B) and 5(A and B)). Vascular surgery was immediately consulted, and the patient was taken emergently to the operating room for an open popliteal artery thrombectomy. Two medium sized clots, and 1 small sized clot were evacuated successfully (Fig. 6).

**Figure 3:**
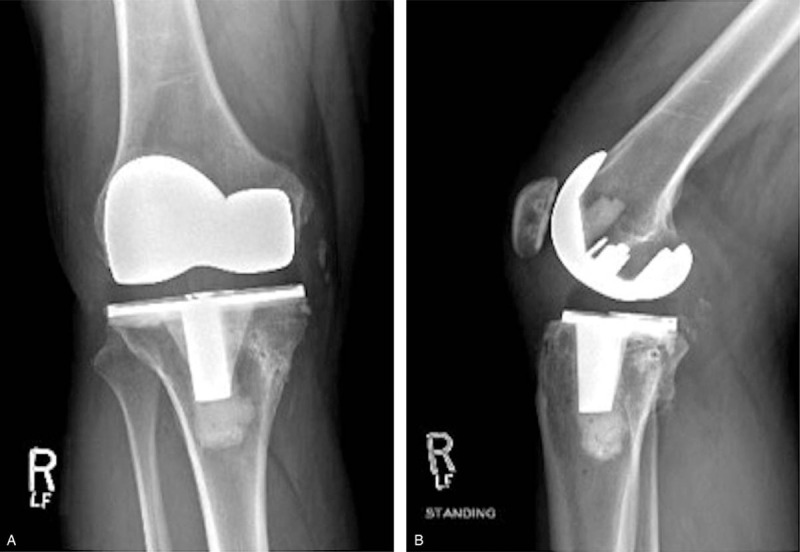
3A: Postoperative AP x-ray demonstrating excellent fixation of the R-TKA. 3B: Lateral x-ray of Figure 3A.

**Figure 4:**
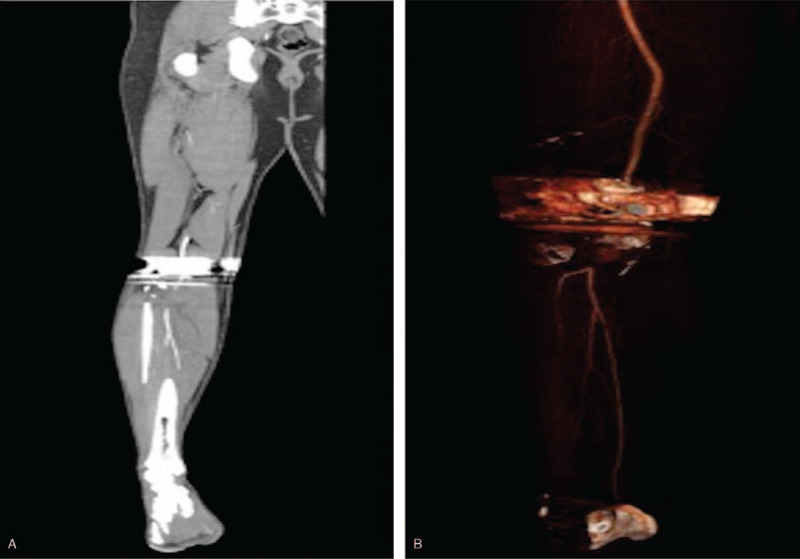
4A-B: Postoperative CT-A demonstrating a thrombus in the distal popliteal artery, inferior to the implant.

**Figure 5:**
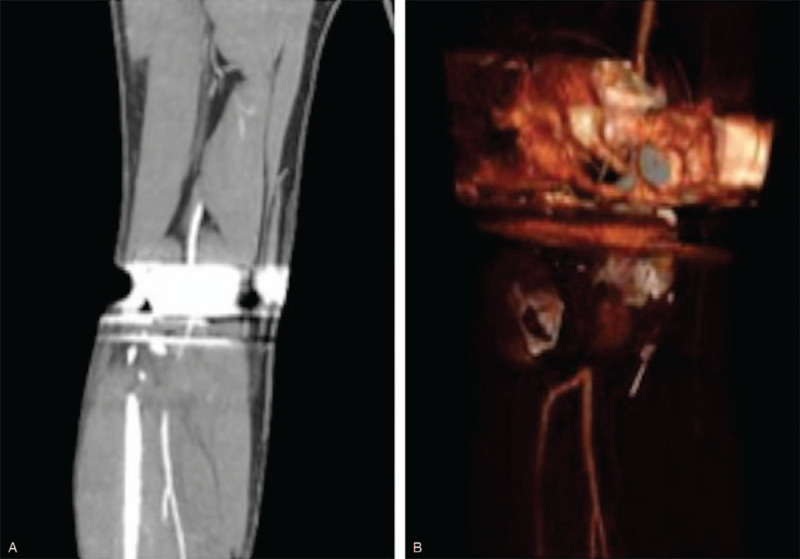
5A-B: Enlarged CT-A images demonstrating clot.

**Figure 6:**
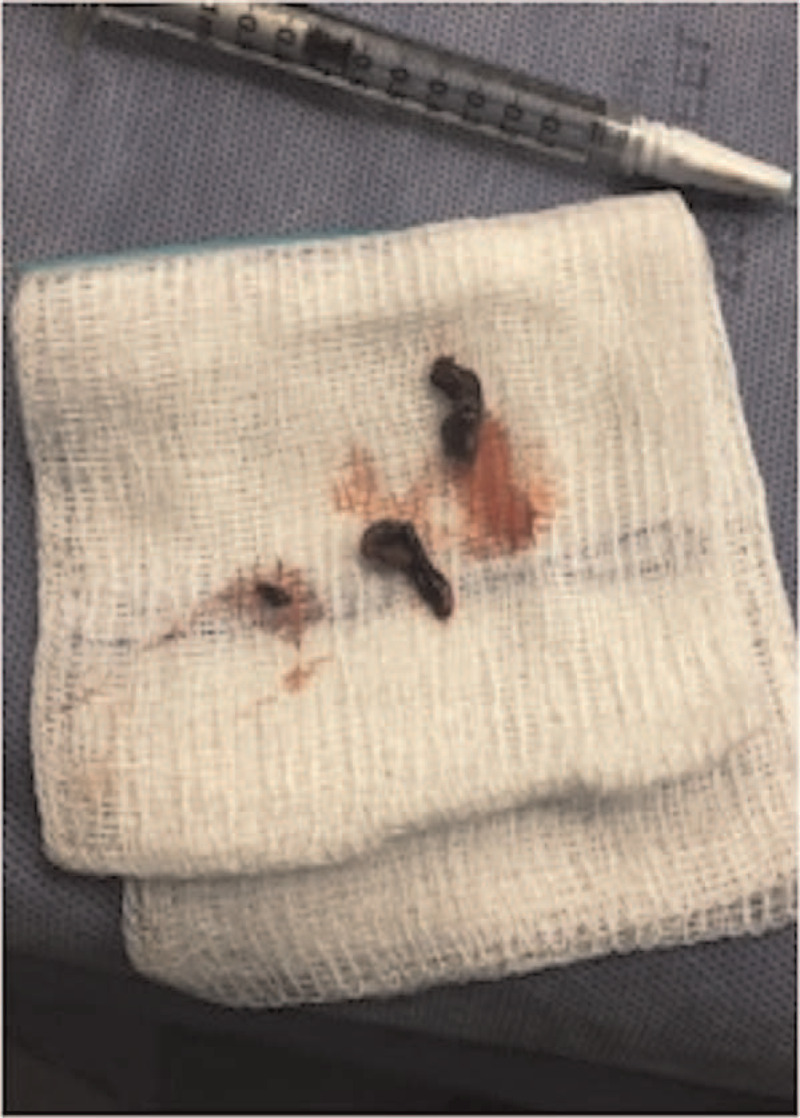
Gross thomboemboli evacuated from the distal popliteal artery by vascular surgery.

## 6 Follow up and clinical outcomes

At one year postoperatively, the patient is doing well with no further complications.

## 7 Discussion

Popliteal artery thrombus following TKA is a rare, but serious complication that can result in limb amputation or death.^[4]^ Significant risk factors, specific to popliteal artery injury following TKA, include: revision surgery, peripheral vascular disease, renal failure, coagulopathy, and metastatic cancer.^[3]^ General thrombotic risk factors include trauma or fractures, major orthopedic surgery, hypercoagulability, previous thrombotic event, age, and metabolic syndrome.^[5]^ A number of other case reports corroborate these risk factors. Tsujimoto et al observed an acute popliteal artery occlusion in an 83-year-old male following revision TKA.^[6]^ Inomata et al describe an acute arterial occlusion after bilateral TKAs in an 80-year-old Asian female.^[7]^ Ohira et al described an acute popliteal thrombus in a 71-year-old male with calcium pyrophosphate dihydrate crystal deposition disease.^[8]^ Matziolis et al reported popliteal thrombus in a healthy 69-year-old female with a BMI of 35, and a 74-year-old female with a history of DVT in the contralateral leg after TKA.^[9]^

Arterial vasospasm is the most likely mechanism of thrombus formation in this patient, which could have occurred through different mechanisms. One is from direct trauma that leads to frank disruption of the popliteal artery, causing severe spasm, and posing a high risk for thrombus induction at the site of injury. Pal et al identified a case in which laceration of the popliteal artery lead to spasm, thrombosis, and subsequent distal collapse.^[10]^ An alternative cause of arterial vasospasm is arterial compression by surrounding soft tissue from manual manipulation during surgery which has been reported to cause acute arterial occlusion.^[6]^ During the case highlighted in this report, there was no significant bleeding that would suggest direct injury to the popliteal artery when the tourniquet was taken down intra-operatively. Additionally, the vascular surgeon did not note any obvious direct injury to the popliteal artery. We, therefore, believe that soft tissue compression from manual manipulation during the surgery may have led to arterial spasm, and ultimately to the formation of the acute thrombus.

Our patient's risk for thrombus formation was likely increased given his prior trauma (tibial plateau fracture), and his history of multiple surgeries (1 ORIF, 2 arthroscopies, and 1 hardware removal) also likely contributed to thrombus formation. It is well documented that revision TKAs carry an increased risk of popliteal injury.^[4]^ No studies have examined the risk of developing a popliteal artery occlusion after primary TKA in patients with a history of non-arthroplasty knee surgeries such as ORIF, arthroscopy, or hardware removal. These surgeries may have increased the patient's risk of developing a thrombus.

Inomata et al described the risk factors in a patient with an acute popliteal artery thrombus following TKA by utilizing Virchow's triad: intravascular stasis (no motion of lower extremities under spinal anesthesia, tourniquet use), arterial damage (anterior displacement of tibia during surgery), and hypercoagulability (obesity).^[7]^ Intravascular stasis, secondary to tourniquet use, is a potential cause for clot development. Tourniquet utilization in TKA has been long debated, and although tourniquets provide decreased perioperative blood loss, there is controversy regarding their role in DVT development.^[11]^ Surgeons should make an informed decision to determine if a tourniquet is appropriate. The majority of popliteal vascular injuries are due to direct trauma to the artery.^[12]^ TKA requires abnormal positioning of the knee to adequately place the knee prosthesis. Hyperextension may occur during preparation of the patella, causing a dramatic tenting of the popliteal artery over the posterior joint line, putting the artery at risk.^[13]^ Furthermore, anterior displacement of the tibia during tibial cementing, and large posterior osteophytes may be significant risk factors perioperatively for popliteal artery injury. Risk of damaging the popliteal artery is significantly increased in revision TKAs, likely due to the heightened surgical correction required for stiffened or distorted soft tissue, leading to excessive tensioning and potential for arterial kinking or transection.^[4]^ Reviewing preoperative x-rays to identify large posterior osteophytes would be beneficial in surgical planning. Perioperatively, care should be taken to avoid excessive forces that may cause kinking of the popliteal artery, especially during tibial preparation and cementing. BMI is a weak risk factor for thrombus formation; however, following a traumatic event such as TKA, obese patients are hypercoagulable compared to similarly-injured normal weight counterparts.^[5]^ BMI was independently associated with an 85% increased risk of developing a thromboembolic complication after injury.^[14]^ Yang et al. described that patients who are obese are twice as likely to form venous thromboemboli compared to patients with a normal BMI (<24.9).^[15]^ Based on these studies, the patient's BMI of 37.53 may have put him at a mildly increased risk for a thromboembolic event.

Young age and a relatively benign past medical history would have placed this patient in low category for thromboembolic risk. However, due to prior trauma, past surgical history, consequent distorted soft tissue and elevated BMI, the patient may have been at a slightly increased risk. Avoidance of tourniquet utilization, preoperative radiograph review, heightened perioperative awareness, and patient optimization, are factors that would potentially improve the already low risk of popliteal artery occlusion following TKA. Mechanistically, in this case, it appears that soft tissue compression may have caused arterial vasospasm, leading to thrombus formation. Further research should focus on validating this mechanism and exploring the role of prior trauma and surgery as risk factors. If a popliteal thrombus develops following TKA, prompt recognition and evacuation with surgery instead of conservative treatment is associated with better outcomes.^[16]^

To our knowledge this is the youngest (38 years old) reported case of an acute popliteal artery occlusion after TKA. Intraoperative arterial vasospasm caused by compression of surrounding soft tissues is the likely mechanism of thrombus formation. Prior trauma, surgical interventions, and obesity, are possible risk factors identified in this patient. Minimizing preoperative risk factors, patient education on the risks/benefits/alternatives of surgery, and careful monitoring, are essential to managing risk and resolving any complications associated with TKA.

## 8 Informed consent

Written informed consent for this case report was obtained from the patient, thus, an ethics review committee or institutional review board was not necessary.

## Author contributions

XXXX.

## References

[1] Healy W Della Valle C Berend K Complications of total knee arthroplasty: standardized list and definitions of the Knee Society. Clin Orthop Relat Res 2013;471:215–20. 22810157 10.1007/s11999-012-2489-y PMC3528930

[2] Chikkanna J Sampath D Reddy V Popliteal artery thrombosis after total knee replacement: an unusual complication. J Clin Diagn Res 2015;9:RJ101–2. 10.7860/JCDR/2015/15434.6712 PMC4668492 26673407

[3] Ko L Dehart M Yoo J Popliteal artery injury associated with total knee arthroplasty: trends, costs and risk factors. J Arthroplasty 2014;29:1181–4. 24556111 10.1016/j.arth.2014.01.007

[4] Ogawa H Matsumoto K Ito Y Indirect popliteal artery transections in revision total knee arthroplasty: a case report. Bull Hosp Joint Dis 2016;72:168–71. 27281324

[5] Previtali E Bucciarelli P Passamonti S Risk factors for venous and arterial thrombosis. Blood Transfus 2011;9:120–38. 21084000 10.2450/2010.0066-10 PMC3096855

[6] Tsujimoto R Matsumoto T Takayama K Acute popliteal artery occlusion after revision total knee arthroplasty. Case Rep Orthop 2015;2015:672164. 26357582 10.1155/2015/672164 PMC4556868

[7] Inomata K Sekiya I Nakamura T Acute arterial occlusion after total knee arthroplasty: a case report. Clin Case Rep 2017;5:1376–80. 28781862 10.1002/ccr3.1075 PMC5538231

[8] Ohira T Fujimoto T Taniwaki K Acute popliteal artery occlusion after total knee arthroplasty. Arch Orthop Trauma Surg 1997;116:429–30. 9266058 10.1007/BF00434007

[9] Matziolis G Perka C Labs K Acute arterial occlusion after total knee arthroplasty. Arch Orthop Trauma Surg 2004;124:134–6. 14658074 10.1007/s00402-003-0602-0

[10] Pal A Clarke J Cameron A Case series and literature review: popliteal artery injury following total knee replacement. Int J Surg 2010;8:430–5. 20452472 10.1016/j.ijsu.2010.04.008

[11] Fukuda A Hasegawa M Kato K Effect of tourniquet application on deep vein thrombosis after total knee arthroplasty. Arch Orthop Trauma Surg 2007;127:671–5. 17102960 10.1007/s00402-006-0244-0

[12] Silva M Sobel M Popliteal vascular injury during total knee arthroplasty. J Surg Res 2003;109:170–4. 12643860 10.1016/s0022-4804(02)00088-4

[13] Ninomiya J Dean J Goldberg V Injury to the popliteal artery and its anatomic location in total knee arthroplasty. J Arthroplasty 1999;14:803–9. 10537254 10.1016/s0883-5403(99)90029-3

[14] Kornblith L Howard B Kunitake R Obesity and clotting. J Trauma Acute Care Surg 2015;78:30–8. 25539200 10.1097/TA.0000000000000490 PMC4279446

[15] Yang G De Staercke C Hooper W The effects of obesity on venous thromboembolism: a review. Open J Prevent Med 2012;2:499–509. 10.4236/ojpm.2012.24069 PMC4520798 26236563

[16] Li Z Xiang S Bian Y Diagnosis and treatment of arterial occlusion after knee arthroplasty: the sooner, the better. Orthop Surg 2019;11:366–72. 31243922 10.1111/os.12494 PMC6595109

